# Biogenic amorphous silica as main driver for plant available water in soils

**DOI:** 10.1038/s41598-020-59437-x

**Published:** 2020-02-12

**Authors:** Jörg Schaller, Andreas Cramer, Andrea Carminati, Mohsen Zarebanadkouki

**Affiliations:** 1 Environmental Geochemistry, Bayreuth Center for Ecology and Environmental Research (BayCEER), University Bayreuth, Universitätsstraße 30, 95447 Bayreuth, Germany; 2 Leibniz Centre for Agricultural Landscape Research (ZALF), 15374 Müncheberg, Germany; 3 Chair of Soil Physics, University Bayreuth, Universitätsstraße 30, 95447 Bayreuth, Germany

**Keywords:** Ecology, Ecology, Environmental sciences

## Abstract

More frequent and longer drought periods are predicted threatening agricultural yield. The capacity of soils to hold water is a highly important factor controlling drought stress intensity for plants. Biogenic amorphous silica (bASi) pools in soils are in the range of 0–6% and are suggested to help plants to resist drought. In agricultural soils, bASi pools declined to values of ~1% or lower) due to yearly crop harvest, decreasing water holding capacity of the soils. Here, we assessed the contribution of bASi to water holding capacity (WHC) of soil. Consequently, ASi was mixed at different rates (0, 1, 5 or 15%) with different soils. Afterwards, the retention curve of the soils was determined via Hyprop method. Here we show that bASi increases the soil water holding capacity substantially, by forming silica gels with a water content at saturation higher than 700%. An increase of bASi by 1% or 5% (weight) increased the water content at any water potential and plant available water increased by up to > 40% or > 60%, respectively. Our results suggest that soil management should be modified to increase bASi content, enhancing available water in soils and potentially decreasing drought stress for plants in terrestrial ecosystems.

## Introduction

Drought is a main issue in terms of terrestrial ecosystem performance and crop production^1–3^. Drought risks are suggested to increase in the future on the continental and the global scale due to climate change^4,5^, threatening agricultural yield and ecosystem performance^6^. During longer drought periods the soil water storage decreases to values at which water is no longer available for plants, leading to severe drought stress and wilting^7^. A key function controlling the plant available water content in soils is the water retention curve (WRC), which describes the capacity of soils to hold water at different water potentials^8^. Soils differ quite substantial in WRC^9^ and agricultural intensification was shown to reduce soil water holding capacity^10^.

Soil biogeochemistry, in particular, soil organic matter content, influences the WRC of soils, with a positive correlation between soil organic matter content and water holding capacity^11,12^. However, less is known about other important biogeochemical processes affecting WRC. It was suggested that silica (Si) fertilization may help plants to survive drought^13,14^, but underlying mechanisms are not clear, yet. Currently, agricultural practice is decreasing the biogenic amorphous silica (bASi) content of soils^15–17^ due to of yearly extractions of bASi by crop harvest^17^, because many crop plants are Si accumulators^18^. Biogenic ASi is present in soils besides other silicon-containing compounds, such as silicates, quartz, or clay^19^. The bASi pool in soils includes phytogenic, zoogenic, microbial, and protozoic Si fractions, with the phytogenic pool being the most frequent in terms of quantity^20,21^. The phytogenic ASi pool consists of pure phytoliths and other amorphous forms of silica like the Si double layer, as a result of silicic acid uptake and sequestration in the plant biomass. This bASi returns back to the soil by littler fall and litter decomposition. For soils from different climate regions, different parent material and different vegetation forms, a large range of bASi content in soils (0.1 to ~ 6%) was found^22^.

There is sparse literature suggesting that ASi addition to soils could potentially increase the water holding capacity of soils^23^, in some cases dramatically to a value of ~ 500%^24^. However, a comprehensive picture of the effect of ASi content on water holding capacity of soils is still missing.

In summary, drought stress of plants (due to low soil water availability) is a main issue for terrestrial ecosystem performance and global agricultural yield. The soil ASi content was suggestd to be positively related to the soil water holding capacity of soils and with this the amount of plant available water. However, no study has analyzed the interdependency between soil ASi content and soil water holding capacity, yet. Therefore, the aim of this study is to determine the importance of soil ASi contents for water holding capacity and water supply to plants. In the context of the highly different amounts of bASi as the main part of soil ASi pools, of the declining contents of these silica pools in agricultural soils and their putative importance for the soil water holding capacity, we analyzed the effect of bASi on WRC of different soils. Specifically, we show the importance of bASi for soil WRC in relation to important soil minerals (montmorillonite, aluminum hydroxide, and calcium carbonate), other biogeochemical controlled soil compounds (calcium oxalate), and dissolved silica as a dissolution product of bASi. Our hypothesis was that: (i) bASi has is very high water holding capacity and (ii) increasing the soil bASi content will lead to an increase of the soil water holding capacity and plant available water.

## Results and Discussion

### High water holding capacity of amorphous silica

**Figure 1:**
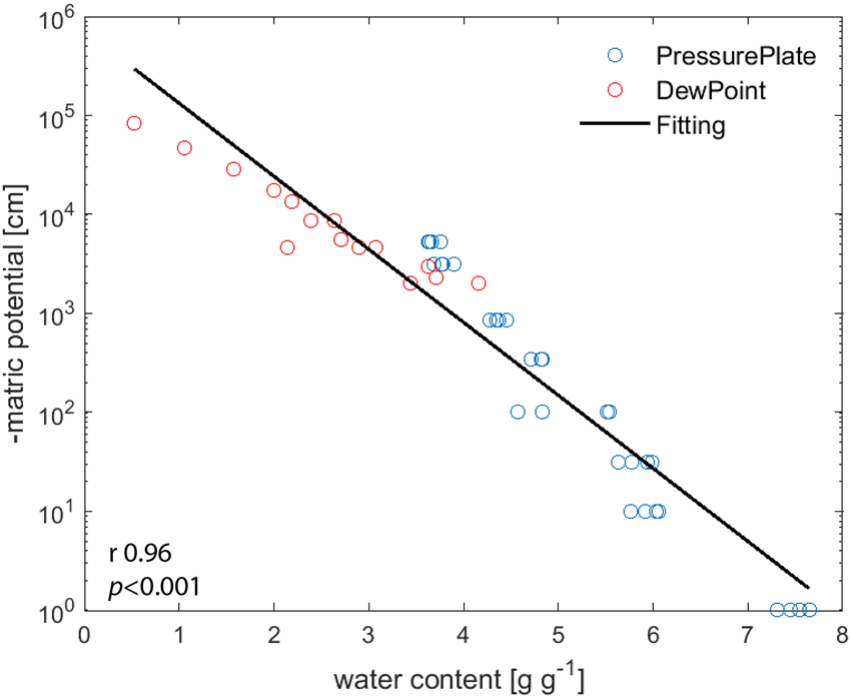
Water potential of biogenic ASi in relation to volumetric water content. The water content of pure bASi as a function of soil matric potential was determined by the combination of pressure plate and dew point measurements. A significant linear relationship was found between the water content and the negative matric potential (Pearson r 0.96, *p* < 0.001, after logarithmic transformation of the matric potential data).

Plant bASi derived from rice straw (with Si concentration of 40 ± 3%) reached a maximum water content greater than 700% (g g^−1^) at saturation and had a water holding capacity (WHC) of 522 ± 12% after centrifuging at 5,000 × g for ten minutes (Fig. S1). The same value (WHC of 515%) was found at a water potential of −10^2^ cm measured by pressure plate apparatus. The WHC found this plant derived ASi is nearly the same as found in the experiments using the artificial analog ASi Aerosil 300 (Evonic, Germany) which was 525 ± 15% (Fig. S1). The maximum water content of this material was the same as for the bASi, reaching values greater than 700% (g g^−1^) at saturation (Fig. 1). The explanation of the equal WHC is that plant ASi and Aerosil 300 have similar surface area (~400 m^2^ g^−1^ for plant bASi and ~300 m^2^ g^−1^ for Aerosil 300) and porosity (0.6–0.9 cm^3^ g^−1^ for plant bASi and 0.56 cm^3^ g^−1^ for Aerosil 300)^25,26^. The high surface area increases the adsorption of water films on the particle surfaces. This high surface area, which is in the range of that of clay minerals^27,28^, combined with the high porosity may lead to the formation of silica gels which are known to have a water holding capacity of more than 500%^29^. As the properties of the plant-derived bASi were comparable to the artificial analog Aerosil 300 in terms of WHC, we used Aerosil 300 as a analog for plant bASi for future experiments, because extraction and purification of the ~1000 g bASi needed for further experiments was impossible due to the large amounts of plant material needed for such extraction. The relation between the volumetric water content and the negative matric potential of bASi revealed a power-law relation (linear relationship, Pearson r 0.96, *p* < 0.001, after logarithmic transformation of the matric potential data) (Fig. 1).

### Amorphous silica amendments strongly increase the soil water holding capacity

**Figure 2:**
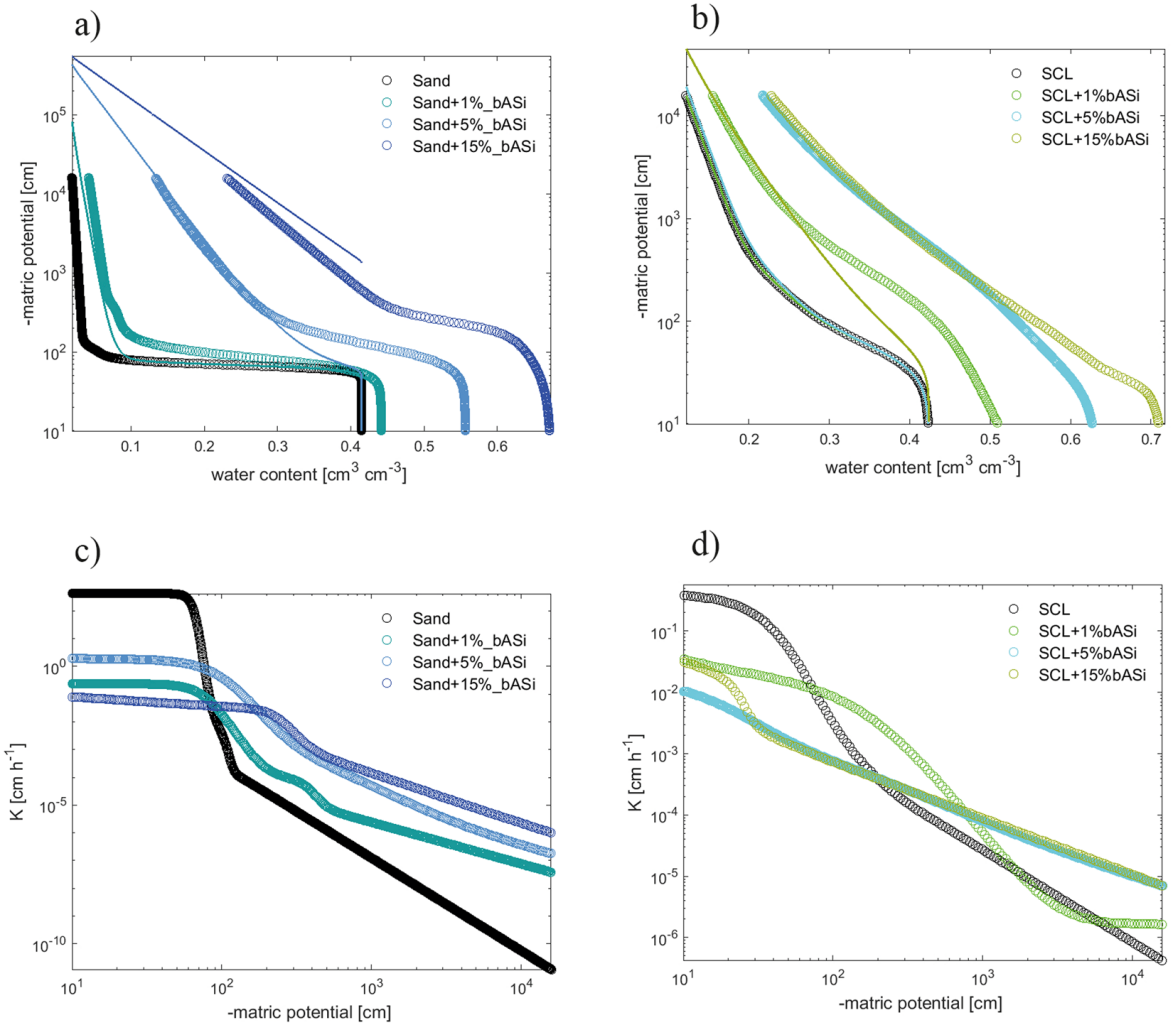
Biogenic ASi increases water holding capacity and soil hydraulic conductivity of soils. Soil water potential as a function of the volumetric water content of sand mixed with different contents of bASi (**a**) and of a sandy clay loam (SCL) mixed with different contents of bASi (**b**). Soil hydraulic conductivity as a function of matric potential of sand mixed with different contents of bASi (**c**) and of a sandy clay loam (SCL) mixed with different contents of bASi (**d**), The dots are the measured retention curves and the lines (**a,b**) are the estimated retention curves based on retention curve of soil without ASi and retention curve of pure bASi.

Increasing the bASi content in soils resulted in a strong increase in soil water content at any water potential (Fig. 2a,b). Biogenic ASi increased the soil water content also at saturation (water potential close to zero). An increase of bASi content by 1%, 5% and 15% (g g^−1^) increased the soil water content at saturation by >3%, >15% and >25%, for both pure sand (Fig. 2a) and the sandy clay loam (SCL) (Fig. 2b), respectively. The greater water content at saturation can be explained by the strong swelling capacity of bASi which increased the soil porosity. The role of bASi in enhancing the water holding capacity of soils can be seen at more negative water potentials. For instance, at a water potential of −10^3^ cm the water content of the control soil was 0.03, while it was 0.08, 0.25 and 0.40 at bASi contents of 1%, 5% and 15% (g g^−1^) for the sandy soil, respectively. The enhanced water retention upon the ASi addition can be explained by the water adsorption capacity of the added bASi. Water in soils is retained by capillary and adsorptive forces^30^. In the pure sand, capillary forces hold water in the soil pores till a matric potential of ca. −80 cm to −100 cm. At more negative water potentials most of the soil water was drained. The enhanced water retention after bASi addition was obtained by adding the adsorptive potential to the matric potential of the control soil. At any given soil water content, the gravimetric water fraction of bASi was calculated and then the corresponding water potential induced by the presence of bASi was estimated from the fit of Fig. 1. This water potential was added to that of the control soil at the same water content. In this calculation it was assumed that bASi does not alter the pore geometry and the capillary forces. As seen in Fig. 2a, this calculation was capable to well reproduce the enhanced water retention due to bASi addition in the sandy soil, particularly at negative water potentials (< −10^3^ cm). However, this calculation strongly underestimated the effect bASi on WRC of the sandy clay loam (Fig. 2b). The fact that the fit was not perfect indicates additional effects on bASi on capillary forces and pore geometry. Indeed, bASi affects the pore geometry by largely increasing the soil porosity at bASi of 5% and 15% (g g^−1^).

### Amorphous silica amendments increase the plant available water in soils

**Figure 3:**
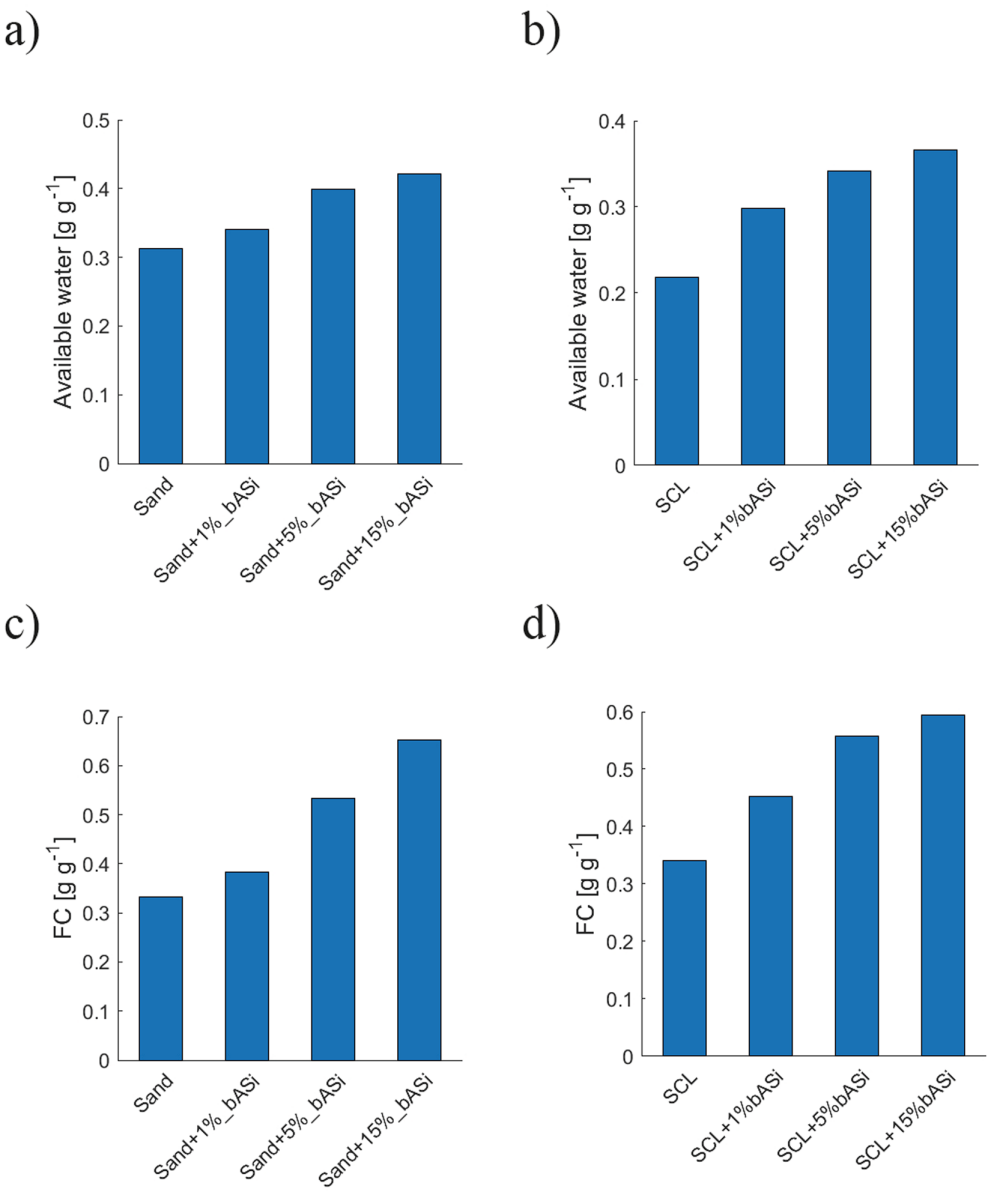
Changes in soil available water and field capacity due to biogenic ASi. Available water of sand mixed with different contents of bASi (**a**), of a sandy clay loam (SCL) mixed with different contents of bASi (**b**) and field capacity of sand mixed with different contents of bASi (**c**), of a sandy clay loam (SCL) mixed with different contents of bASi (**d**). Significant linear relationship were found between available water and sand ASi content (Pearson r 0.92, *p* = 0.04) and SCL ASi content (Pearson r 0.96, *p* = 0.02) as well as between FC and sand ASi content (Pearson r 0.96, *p* = 0.02) and SCL ASi content (Pearson r 0.9, *p* = 0.05).

By altering the WRC, bASi increased the volume of water that is available to plants, the so-called plant available water (AW) (Fig. 3a,b). Such value can be calculated as the water held by the soil between water potentials of −60 cm and −15000 cm. These values correspond approximately to the field capacity (FC) (Fig. 3c,d) and the permanent wilting point (PWP) (Fig. S4a,d)^31^ and are defined as the water that is stored in soils after precipitation and that can be extracted by the plants. The plant available water for the pure sand and for the 1%, 5% and 15% (g g^−1^) bASi addition were 0.31, 0.35, 0.39 and 0.43 g g^−1^, respectively (significant linear relationship between bASi content and plant available water, Pearson r 0.92, *p* = 0.04). Besides increasing AW, the addition of bASi increased water content at permanent wilting point. The PWP for the pure sand and for the 1%, 5% and 15% (g g^−1^) bASi addition were 0.02, 0.04, 0.13, and 0.23 g g^−1^ respectively. When the soil approaches the PWP in such a coarse-textured soil, liquid phase may become fragmented^32^ and microbial activities and nutrient diffusion may drop. The addition of bASi increased the water content at PWP, possibly facilitating microbial activities and diffusion of solutes and nutrients.

Measurements of a sandy clay loam (SCL) showed similar results, with the addition of bASi enhancing the water content, both at saturation and at more negative water potentials. As for the sandy soil, also in the sandy clay loam bASi increased the plant available water, but to even higher values (more than 40% due to addition of 1% bASi and 60% due to addition of 5% bASi) (Fig. S4d). The plant available water for the sandy clay loam and for the 1%, 5% and 15% (g g^−1^) bASi addition were 0.21, 0.3, 0.34 and 0.47 g g^−1^ respectively (significant linear relationship between bASi content and plant available water, Pearson r 0.96, *p* = 0.02).

### The soil silica cycle affects the water holding capacity of soils

The result that bASi increases the plant available water is highly important as agricultural practice tends to decrease soil bASi pools due to yearly bASi export by crop harvest^17^. Hence, soils used for agriculture exhibit very low bASi content (~1% or lower for most soils)^33^. As soil bASi content in soils is in the range of 0 and 6%, the increase by 5% bASi as shown in Fig. 2 is within the natural range of soil bASi pools. Biogenic ASi (mostly phytoliths) can be preserved in soils for many years (as bASi) until Si is mobilized to dissolved Si (DSi) by phytolith dissolution^34^. Increasing DSi concentration in soils slightly decreased the soil WRC (Fig. S2b). On the other hand, high concentrations of DSi can lead to neoformation of clay minerals^20,35^, which are known to have a high water holding capacity^23^. However, the WRC of the soil with 5% (g g^−1^) bASi content is even higher compared with the soil with 5% (g g^−1^) montmorillonite addition (Fig. S2b), a mineral of the fine clay fraction, known to increase the soil water holding capacity. Hence, the speciation of Si is highly important because it determines the WRC of soils.

By enhancing the soil water content at negative water potentials, bASi affects the soil hydraulic conductivity (Fig. 2c,d). The addition of bASi decreased the soil hydraulic conductivity at saturation but it increases its values at negative water potentials. This is particularly evident in the sand, whose drop in conductivity between soil matric potentials of −100 cm to −10000 cm was strongly attenuated by the presence of bASi (Fig. 2c).

### Effect of different soil amendments on water holding capcity

Plants may accumulate not only bASi but also calcium oxalate (Ca-ox) to concentration up to more than 20% dry weight^36^. Accumulation of Ca-ox in soils after plant dieback and litter decomposition leads to an enrichment of soil by Ca-ox. Besides plants, also soil fungi increase the Ca-ox content in the soil, as Ca-ox is a major metabolite of fungi^37^. Soils differ in Ca-ox contents (~ 0 to > 200 mg kg^−1^ DW^−1^^38^). Increasing soil Ca-ox content increased the WRC (Fig. S2a), especially under negative water potentials. However, common field values of Ca-ox in soils (< 1%)^39^ suggest that Ca-ox effects on soil WRC are negligible. An increase of other soil minerals by addition of 5% (g g^−1^) of either calcium carbonate or aluminum hydroxide had only negligible effects on soil WRC, with calcium carbonate slightly increasing and aluminum hydroxide slightly decreasing the soil water content at any water potential (Fig. S2b). Compared to the strong effects of bASi on available water, field capacity and wilting point, the addition of Ca-ox, DSi, calcium carbonate or aluminum hydroxide are much less important for soil water relations (Figs. S3 and S4).

## Conclusion

**Figure 4:**
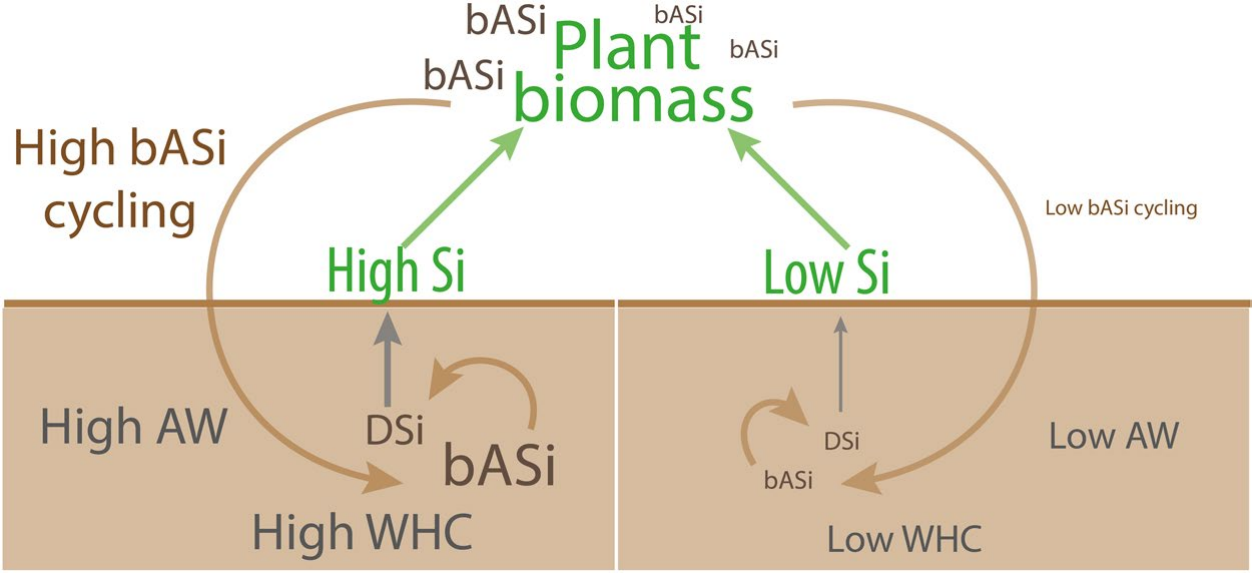
The importance of biogenic ASi cycling on soil water holding capacity. Scheme showing the role of biogenic amorphous silica (bASi) cycling via dissolved silicon (DSi) uptake and bASi cycling via litterfall for water holding (WHC) capacity and available water (AW) of soils.

The effect of bASi on the soil water retention capacity is extremely high compared to that of clay minerals^12^, with bASi strongly increasing WHC. As agricultural practices tend to deplete bASi pools in soils, the water holding capacity of soils exposed to intensive agriculture is expected to continuously decrease. Soils with a high Si availability provide large amounts of Si to plants. Hence, plants may accumulate higher amounts of bASi and recycle more bASi to soils via plant dieback followed by litter decomposition. A higher bASi accumulation in soils will lead to higher water holding capacity and more available water in soils, hence reducing drought stress for plants (Fig. 4). The large effect of the amorphous Si structures with high surface area and porosity on water holding capacity may also explain why Andosols (soil with high tephra content, which are also Si structures with high surface area and porosity) have a very high water holding capacity^40^, in addition to the known effects of allophane^41^. To cope with the predicted increased intentsification of drought periods, potentially strongly affecting ecosystem performance, increasing bASi content of soils may be a way to increase WHC of soils. This increase in soil WHC by increase of soil bASi content may potentially decrease negative effects of drought on ecosystem performance. An increase of the soils “bASi” content can also be achieved by amending soils using purchasable artificial silica with the same properties as bASi.

## Materials and Methods

### Extraction of biogenic amorphous silica

Plant-derived bASi was extracted from rice straw by dry ashing method followed by ten successive extractions with *aqua regia*. These ten successive extractions each used 5 g of plant Si in 100 mL *aqua regia* for one day at room temperature, modified after Parr, *et al.*^42^. Afterward, the material was rinsed with pure water until all added acid was washed out. The material was afterwards analyzed for Si concentration by alkaline extraction and ICP-OES measurements according to DeMaster^43^. As the bASi content constitute only a little fraction of the dry matter of the used plant material (~3%) we could extract only a few g of bASi. The extracted bASi was dried at 40 °C in oven for 48 h.

### Analysis of the water holding capacity of plant derived biogenic silica and its artificial analog

The water content of this extracted bASi was determined as follows: a known quantity of dry bASi was placed in centrifuge vials and mixed with water, for 5 hours. One aliquot of the mixture was centrifuged at 5,000 g for 10 min and the exceeding water was discarded and the sample was weighed again. The other aliquot was analyzed by pressure plate apparatus (Eijkelkamp, Netherlands). The difference between weight of dry sample and then the one after centrifugation was used to quantify maximum water holding capacity of the sample. The same was done for the amorphous silicate Aerosil 300 (Evonic, Germany). Each experiment was performed with five replications.

In parallel experiments the retention curve (relation between soil water content and soil water potential) of pure bASi treatment was determined by combination of hanging column method, pressure plate apparatus (Eijkelkamp, Netherlands) and dew point tensiometer (Meter Group, Germany). The hanging column method was used for water potentials of −1, −10, −31, and −100 cm, the pressure plate method for water potentials of −340 −850, −3130 and −5250 cm. For these measurements, pure bASi was pre-saturated with water for 48 h and then afterward equilibrated with different water potential. Equilibrium was assumed to be reached when no water was flowing out of the samples for at least two successive days. When equilibrium was reached, the samples were weighed, and the water content was gravimetrically determined. For lower water potential, a dew point tensiometer (WP4C, Meter Group, Germany) was used to determine water potential at drier range. The pure bASi was adjusted to different water contents and let to equilibrate for two days (no evaporation was occurring during this time. Afterwards, the soil water potential was measured using the WP4C and the soil water content was determined gravimetrically.

### Experimental design and analysis

The treatments for the experiments to determine the water retention curve were: quartz sand with 0, 1, 5, or 15% (weight) the artificial bASi (Aerosil 300, Evonic), quartz sand with 5% montmorillonite (naturally occurring, 200 nm mesh powder, Alfa Aesar, as naturally occurring clay mineral), quartz sand with 5% dissolved silica (DSi) (sodium metasilicate nonahydrate, >98%, Sigma-Aldrich, which dissolves into DSi), quartz sand with 5% aluminum hydroxide (fine powder, pure hydrargillite, Merck), quartz sand with 5% calcium carbonate (powder < 30 µm particle size, >98% pure, Aldrich), and quartz sand with three different levels of calcium oxalate (Ca-ox; calcium oxalate monohydrate, fine powder, > 98% pure, Carl Roth GmbH & Co KG) (in the same concentration level as for bASi; 1, 5, or 15%, for better comparability). Additionally, we used a sandy clay loam (SCL) mixed with 0, 1, 5, or 15% of the artificial bASi. The soil mixture was prepared with mixing air-dried soils with different amendments. The pure sand had a particle size in the range between 100 and 200 µm, a pH of 5.7 (measured in CaCl_2_ solution), an electric conductivity of 4 µS cm^−1^ and no other mineral or organic matter inside. The sandy clay loam had particle content of 64.2% sand, 3.88% silt and 31.92% clay, a pH of 7 (measured in CaCl_2_ solution), an electric conductivity of 234 µS cm^−1^ and organic matter content of 2.9%.

The effect of the different additions on water-related properties of soil such as soil retention and soil hydraulic conductivity curves was determined using an evaporation measurement device (HYPROP meter group, Germany).

HYPROP system was used to measure soil matric potential at two different locations with an interval of 2.5 cm, the average soil water content and the evaporative flux during soil drying cycle via evaporation^44^. From the gathered data (soil water potentials and soil water contents) we calculated the soil available water, the permanent wilting point, and field capacity.

## Supplementary information

Supplementary Information.
