# Supplementary material for: Biogenic amorphous silica as main driver for plant available water in soils
Source: Sci Rep. 2020 Feb 12;10:2424. doi: 10.1038/s41598-020-59437-x (PMC7016105; doi:10.1038/s41598-020-59437-x)
Supplement: Supplementary file 1 — Supplementary Information. [file 41598_2020_59437_MOESM1_ESM.pdf]

Supplementary Information to:

Biogenic amorphous silica as main driver for plant available water in soils

Authors

Jörg Schaller[1], Andreas Cramer[1,2], Andrea Carminati[2], and Mohsen Zarebanadkouki[2]*

Affiliations

[1] Environmental Geochemistry, Bayreuth Center for Ecology and Environmental Research (BayCEER), University Bayreuth, Universitätsstraße 30, 95447 Bayreuth, Germany

[2] Chair of Soil Physics, University Bayreuth, Universitätsstraße 30, 95447 Bayreuth, Germany

Supplementary Figures

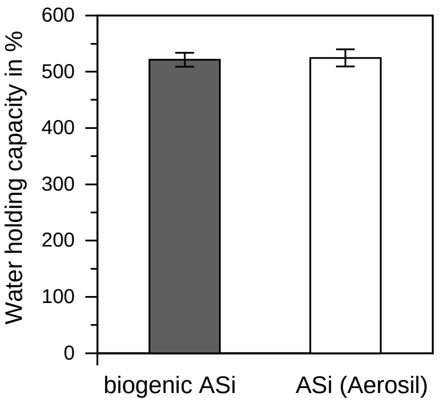

Fig. S1. High water holding capacity of biogenic amorphous silica and its artificial analog. Water holding capacity after centrifuge at 5,000 ×g of biogenic ASi derived from rice straw and of the artificial analog (the amorphous silica Aerosil).

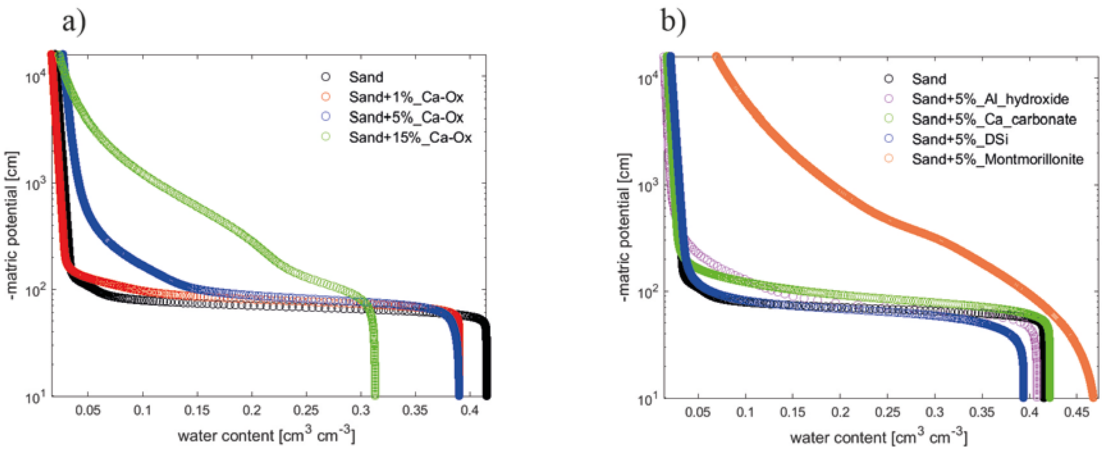

Fig. S2. Water holding capacity of sand with different amendments. Soil water potential as function of volumetric water content of soil mixed with different contents of calcium oxalate (a) and of sand mixed with 5% of aluminum hydroxides (Al_hydroxide), calcium carbonate (Ca_carbonate), dissolved silica (DSi), or montmorillonite, each (b).

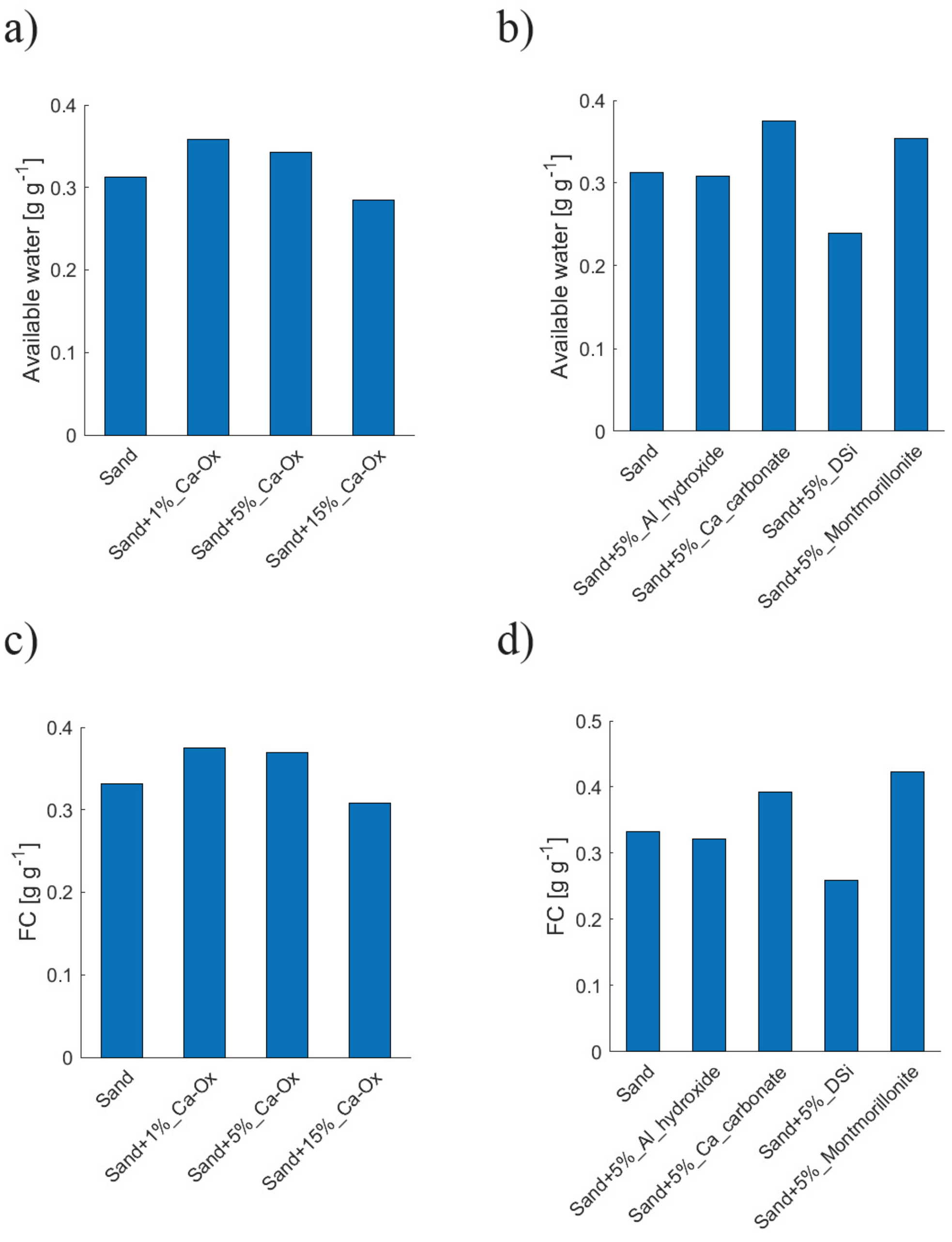

Fig. S3. Soil available water and field capacity of sand being affected by calcium oxalate, aluminum hydroxid, calcium carbonate, dissolved silica (DSi) and montmorillonite. Available water of sand mixed with different contents of calcium oxalate (Ca-ox) (a) and sand mixed with 5% of aluminum hydroxides (Al_hydroxide), calcium carbonate (Ca_carbonate), dissolved silica (DSi), or montmorillonite, each (b). Field capacity of sand mixed with different contents of calcium oxalate (Ca-ox) (c) and sand mixed with 5% of aluminum hydroxides (Al_hydroxide), calcium carbonate (Ca_carbonate), dissolved silica (DSi), or montmorillonite (d).

37

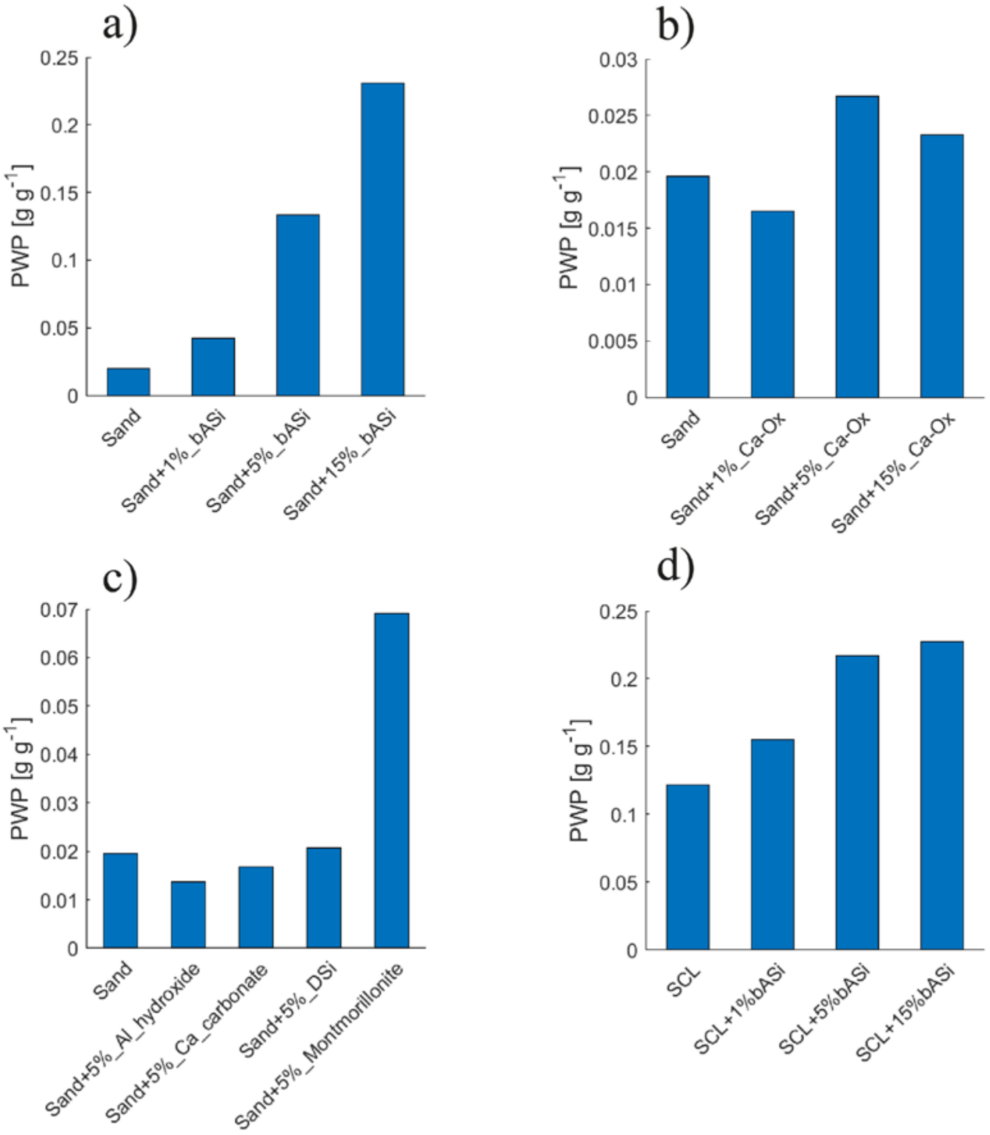

Fig. S4. Permanent wilting point of sand and silty clay loam being affected by bASi, aluminum hydroxid, calcium carbonate, dissolved silica (DSi) and montmorillonite. Permanent wilting point of sand mixed with different contents of bASi (a), of sand mixed with different contents of calcium oxalate (Ca-ox) (b), of sand mixed with 5% of aluminum hydroxides (Al_hydroxide), calcium carbonate (Ca_carbonate), dissolved silica (DSi), or montmorillonite, each (c), and of a sandy clay loam (SCL) mixed with different contents of bASi (d).
